# 5.3 EVIDENCE ON A TRANSDIAGNOSTIC PSYCHOSIS SPECTRUM OF SCHIZOPHRENIA, SCHIZOAFFECTIVE AND PSYCHOTIC BIPOLAR DISORDER IN THE BIPOLAR-SCHIZOPHRENIA NETWORK ON INTERMEDIATE PHENOTYPES (B-SNIP)

**DOI:** 10.1093/schbul/sby014.015

**Published:** 2018-04-01

**Authors:** Ulrich Reininghaus, Jan Boehnke, UnYoung Chavez-Baldini, Brett Clementz, Godfrey Pearlson, Matcheri Keshavan, John Sweeney, Carol Tamminga

**Affiliations:** 1 Maastricht University; 2 University of Dundee; 3 University of Georgia; 4 Yale University; 5 Harvard University; 6 University of Texas Southwestern; 7 The University of Texas Southwestern Medical Center

## Abstract

**Background:**

The validity of the classification of non-affective and affective psychoses as distinct entities has recently been disputed in light of calls for a dimensional and transdiagnostic approach to diagnostic classification and evidence on shared aetiological factors. Despite the shifts in view, there remains a dearth of empirical efforts to clarify and identify a transdiagnostic spectrum of psychosis. Our recent research has demonstrated evidence for a transdiagnostic psychosis spectrum as detailed in a bifactor model with one transdiagnostic symptom dimension and five specific symptom dimensions of positive symptoms, negative symptoms, disorganization, mania, and depression in patients with schizophrenia, schizoaffective and bipolar disorder. The aim of the current study was to investigate whether there is a transdiagnostic dimension cutting across symptoms of schizophrenia, schizoaffective disorder and psychotic bipolar I disorder using widely established measures for assessing psychosis, mania and depression in the large multi-centre Bipolar-Schizophrenia Network on Intermediate Phenotypes (B-SNIP) consortium in the United States.

**Methods:**

This study analysed data from the B-SNIP Phenotyping Consortium, which included 933 patients with a diagnosis of schizophrenia (n=397), schizoaffective disorder (n=224), and bipolar disorder (n=312). Multidimensional item-response modelling was conducted on symptom ratings of the Positive and Negative Syndrome Scale (PANSS), the Young Mania Rating Scale (YMRS), and the Montgomery-Åsberg Depression Rating Scale (MADRS) using the mirt package of the R environment.

**Results:**

A bifactor model with 1 transdiagnostic symptom dimension and 5 specific symptom dimensions of positive symptoms, negative symptoms, cognitive disorganization, mania, and depression best matched the B-SNIP sample data. The bifactor model with 1 transdiagnostic factor and 5 specific factors based on the PANSS 5-factor solution by Emsley et al. (2003) provided the best model fit (AIC=53209.8, BIC=53920.0, aBIC=53443.7), as compared with a unidimensional model (AIC=55583.1, BIC=56151.3, aBIC=55770.2), a pentagonal model based on the PANSS 5-factor solution by Emsley et al.3 (AIC=53452.6, BIC=54068.1, aBIC=53655.3) as well as pentagonal and bifactor models of other previously reported factor solutions. When we extended analyses to include YMRS and MADRS, again, the bifactor model with 1 transdiagnostic factor and 5 specific factors, again, provided the best model fit.

**Discussion:**

Consistent with our previous findings, this study provides evidence on a transdiagnostic symptom dimension that cuts across traditional diagnostic boundaries of schizophrenia, schizoaffective disorder and psychotic bipolar disorder using three widely established measures for assessing psychosis, mania and depression. The best-fitting, bifactor model also included 5 specific symptom dimensions based on the PANSS 5-factor solution by Emsley et al. (2003), which reflects a direct replication of our previous findings on the dimensionality of the PANSS. Overall, our findings lend further support to a transdiagnostic psychosis spectrum encompassing schizophrenia, schizoaffective and bipolar disorder as we have previously proposed.

